# Supplementary material for: Cardiac radiotherapy induces electrical conduction reprogramming in the absence of transmural fibrosis
Source: Nat Commun. 2021 Sep 24;12:5558. doi: 10.1038/s41467-021-25730-0 (PMC8463558; doi:10.1038/s41467-021-25730-0)
Supplement: Supplementary file 1 — Supplementary Information [file 41467_2021_25730_MOESM1_ESM.pdf]

Supplementary Information file for:

Cardiac radiotherapy induces electrical conduction reprogramming in the absence of transmural fibrosis

David M. Zhang[1,2], Rachita Navara[1,2], Tiankai Yin[2], Jeffrey Szymanski[3], Uri Goldsztejn[2,4], Camryn Kenkel[2,4], Adam Lang[5], Cedric Mpoy[3], Catherine E. Lipovsky[2,6], Yun Qiao[2,4], Stephanie Hicks[2], Gang Li[2,4], Kaitlin M.S. Moore[1,2], Carmen Bergom[1,3], Buck E. Rogers[3], Clifford G. Robinson[1,2,3], Phillip S. Cuculich[1,2,3], Julie K. Schwarz[1,3], Stacey L. Rentschler*[1,2,4,6]

[1]Center for Noninvasive Cardiac Radioablation
[2]Department of Medicine, Cardiovascular Division
[3]Department of Radiation Oncology
[4]Department of Biomedical Engineering
[5]Department of Pathology
[6]Department of Developmental Biology
Washington University in St. Louis, School of Medicine
660 S. Euclid Ave. Saint Louis, MO 63110

*Corresponding Author

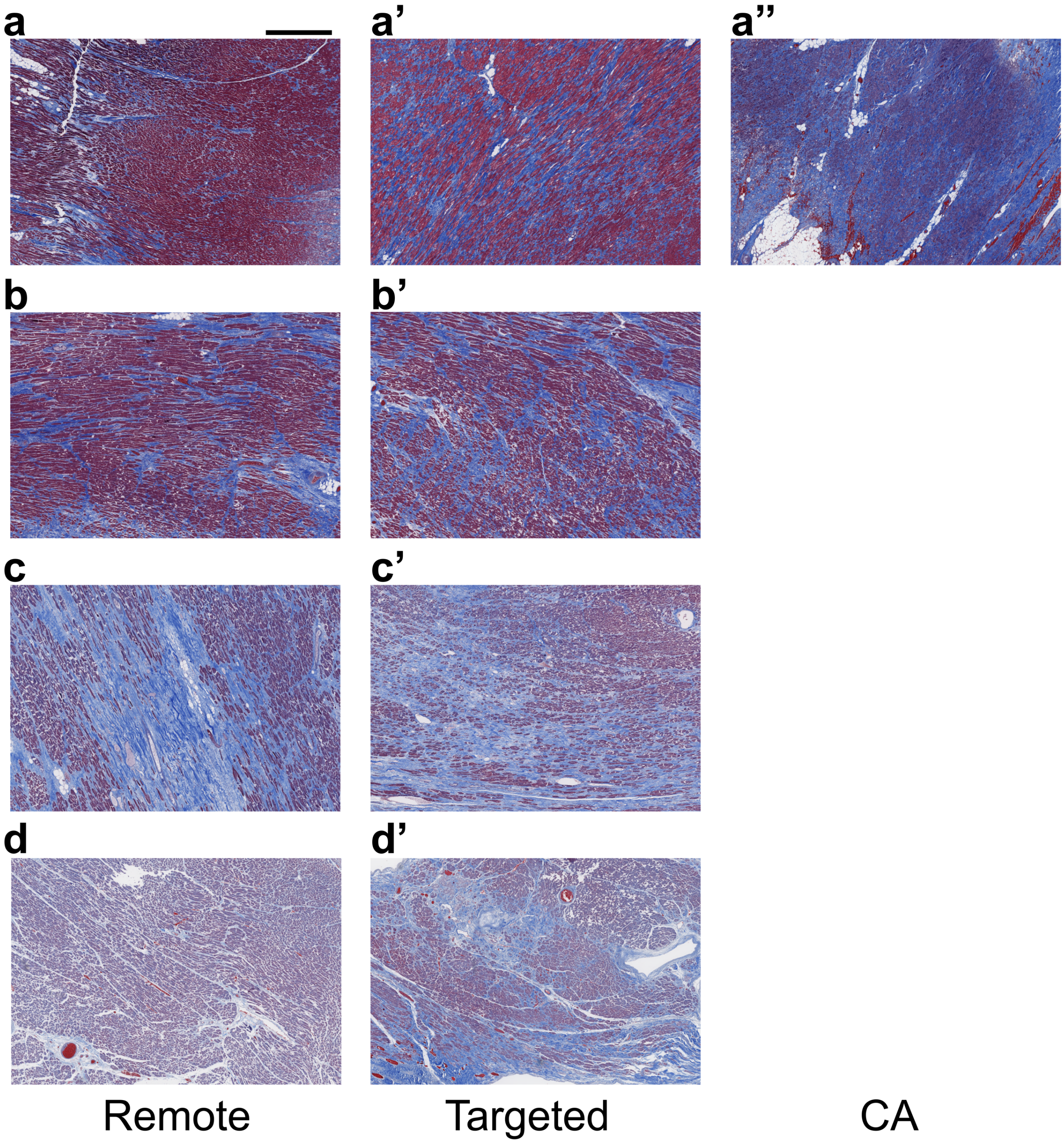

Supplementary Fig. 1. Cardiac RT does not create catheter ablation-like scar. Left column: Nontargeted regions of cardiac RT patient hearts. Middle column: targeted regions of hearts that received 25 Gy ionizing radiation in the same patients for treatment of VT. **(a, a’, a’’)** Representative trichrome images from remote, IR-targeted, and radiofrequency ablation regions of Patient A’s explanted heart, respectively. Patient A received heart transplant 478 days after

RT treatment and previously underwent failed radiofrequency CA. **(b, b')** Representative trichrome images from remote and IR-targeted regions of Patient B's postmortem heart, respectively. Patient B expired 251 days after RT, and heart failure progression is suspected as the cause of death. **(c, c')** Representative trichrome images of remote and IR-targeted regions of Patient C's postmortem heart. Patient C expired 209 days after RT, and the cause of death was chronic amiodarone-induced pulmonary fibrosis. **(d, d')** Representative images of targeted and nontargeted regions of Patient D's postmortem heart. Patient D expired 17 days after therapy due to an acute ischemic stroke. Scale bars = 1 mm.

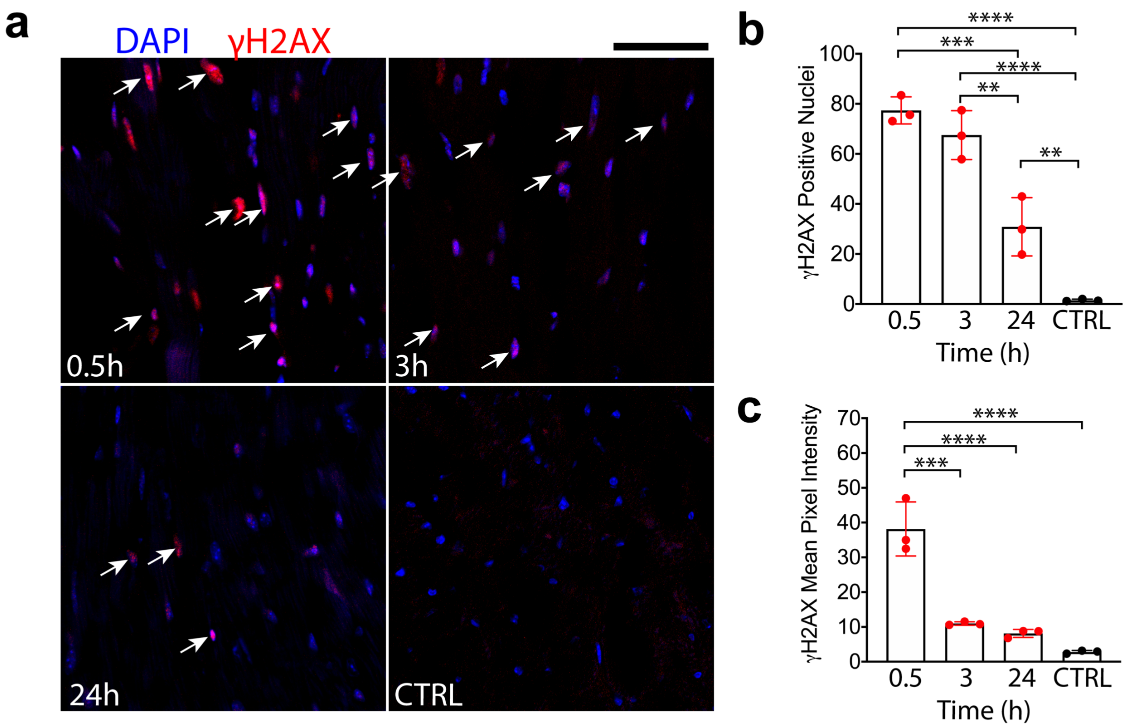

Supplementary Fig. 2. The adult mammalian heart rapidly recovers from radiation-induced DNA damage as detected by γH2AX. (a) Immunostaining of hearts for γH2AX (red) and DAPI (blue) after 25 Gy ionizing radiation at 0.5h, 3h, and 24h post-IR, or sham control. Dual-positive nuclei are highlighted with white arrows. **(b)** Quantification of percent γH2AX-positive nuclei in each irradiated (red) or control (black) condition. *n* = 3 biologically independent specimens per condition; ***P* = 0.0023 (3h vs 24h) and 0.0091 (24h vs CTRL); ****P* = 0.0005 (0.5h vs 24h); *****P* < 0.0001 (0.5h vs CTRL and 3h vs CTRL). **(c)** Quantification of mean γH2AX-foci intensity in each irradiated (red) or control (black) condition. (*n* = 3 biologically independent sample per condition); ****P* = 0.0001 (0.5h vs 3h); *****P* < 0.0001 (0.5h vs 24h and 0.5 vs CTRL). All images were taken with the same microscope settings. Scale bars = 50 µm. Statistical analysis consisted of a one-way ANOVA followed by one-sided Tukey post-hoc test of multiple comparisons. All bar graphs are represented as mean ± SD. Source data are provided as a Source Data file.

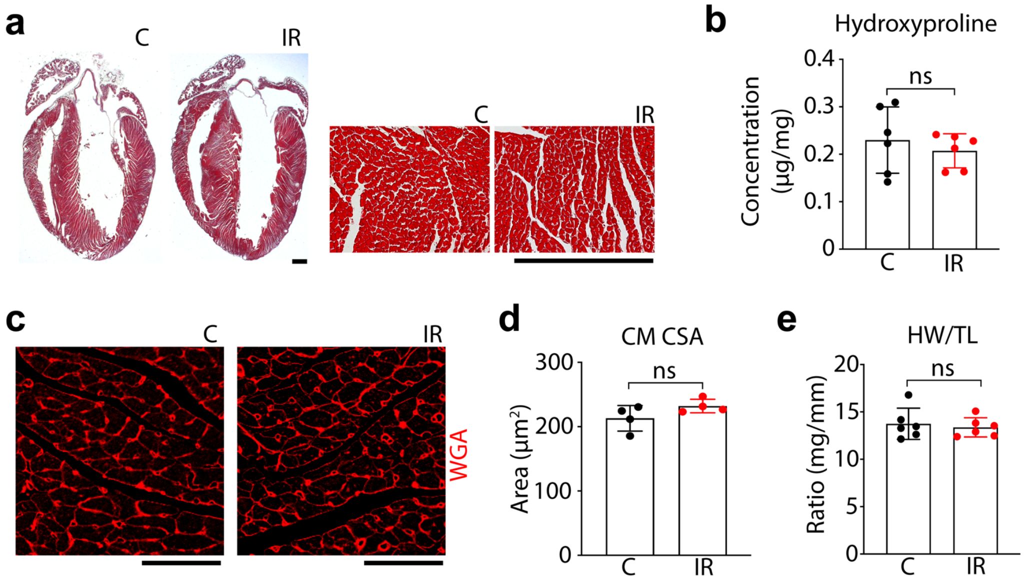

Supplementary Fig. 3. Cardiac RT does not affect gross cardiac morphology at 6 weeks post-IR. (a) Two magnifications of Masson's trichrome stains of control and IR mouse hearts at 6 weeks post-IR. Scale bars = 500 μm. Experiment was replicated three times in biologically independent mice and produced similar results. **(b)** Hydroxyproline content of control and IR hearts after 6 weeks (*n* = 6 biologically independent samples per condition; *P* = 0.50). **(c)** Representative WGA staining of control (left) and IR mouse (right) hearts at 6 weeks post-IR. Scale bars = 100 μm. Experiment was replicated four times in biologically independent mice and produced similar results. **(d)** Quantification of cardiomyocyte cross sectional area at 6 weeks post-IR reveals no difference (*n* = 4 biologically independent specimens per condition; *P* = 0.14). **(e)** Heart weight to tibia length ratios at 6 weeks post-IR (*n* = 6 biologically independent samples per condition; *P* = 0.63). *P* values determined by two-way unpaired *t*-test. All bar graphs are represented as mean ± SD. Source data are provided as a Source Data file.

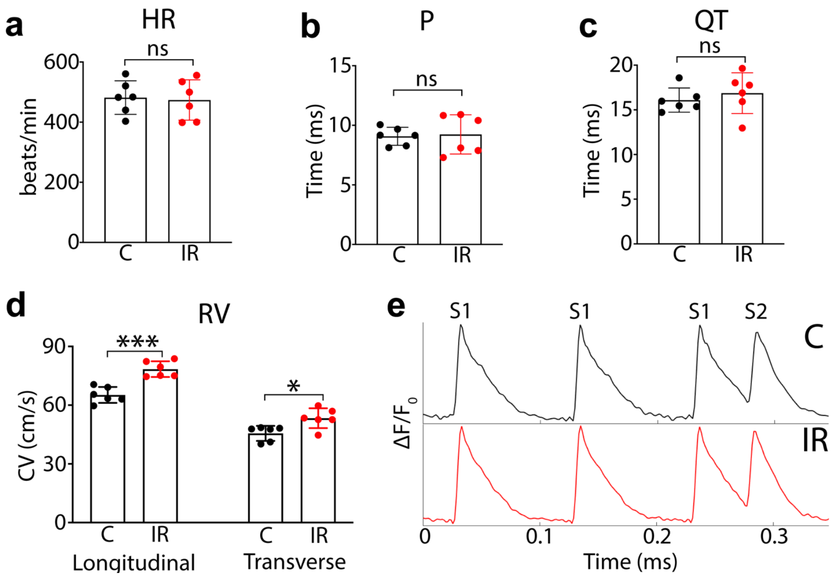

Supplementary Fig. 4. Cardiac IR increases cardiac conduction in mice without affecting repolarization or other rhythm parameters. (a to **c)** Heart rate (HR, *P* = 0.82), P interval (*P* = 0.83), and QT interval (*P* = 0.49) of control versus IR mouse hearts at 6 weeks post-IR as measured by ECG reveal no differences (*n* = 6 biologically independent samples per condition). (**d)** Right-ventricular conduction velocities of control versus irradiated mouse hearts at 6 weeks post-IR (*n* = 6 biologically independent samples per condition; ****P* = 0.00022; **P* = 0.014,) demonstrate similar conduction increases as seen in the left ventricle. (**e**) Representative optical action potentials from control (top) and IR (right) during S1-S2 ventricular stimulation. Representative S1-S2 cycle lengths for each mouse was within 5 ms of recorded ventricular effective refractory periods. *P* values determined by two-way unpaired *t*-test. All bar graphs are represented as mean ± SD. Source data are provided as a Source Data file.

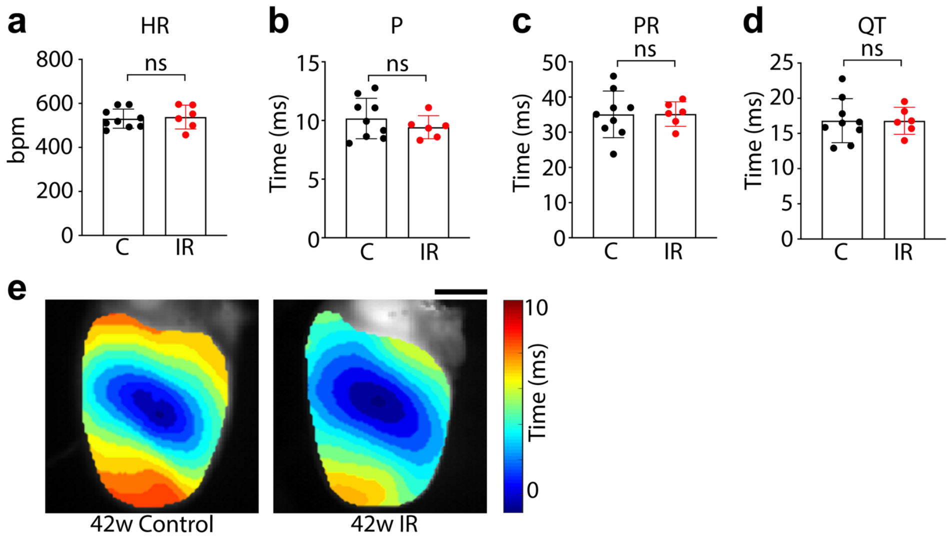

Supplementary Fig. 5. Cardiac IR persistently increases conduction and shortens the QRS interval without affecting other ECG parameters. (**a** to **d**) Heart rate (HR, *P* = 0.77), P interval (*P* = 0.36), PR interval (*P* = 0.97), and QT interval (*P* = 0.99) of control (*n* = 9 biologically independent animals) versus irradiated (*n* = 6 biologically independent animals) mouse hearts at 6 weeks post-IR as measured by ECG . (**e**) Representative ventricular activation maps from control (left) and IR (right) mice 42 weeks post-IR. Scale bars = 3 mm. *P* values determined by two-way unpaired *t*-test. All bar graphs are represented as mean ± SD. Source data are provided as a Source Data file.

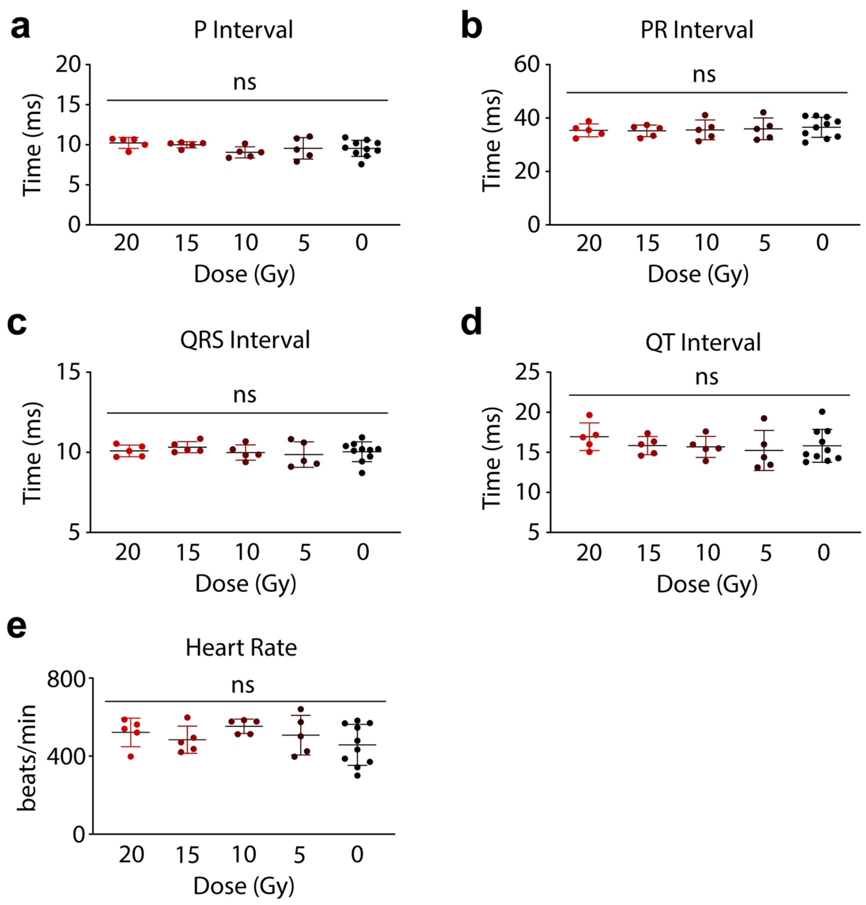

Supplementary Fig. 6. Baseline murine ECG intervals are comparable across treatment conditions. Baseline ECG intervals prior to dose-dependent treatment. **(a)** P interval ($P = 0.31$), **(b)** PR interval ($P = 0.95$), **(c)** QRS interval ($P = 0.69$), **(d)** QT interval ($P = 0.85$), and **(e)** heart rate (HR, $P = 0.34$) at 6 weeks post IR ($n = 5$ biologically independent animals per IR condition; $n = 10$ biologically independent 0 Gy control animals). Nonsignificant statistics determined by ordinary one-way ANOVA. All bar graphs are represented as mean ± SD. Source data are provided as a Source Data file.

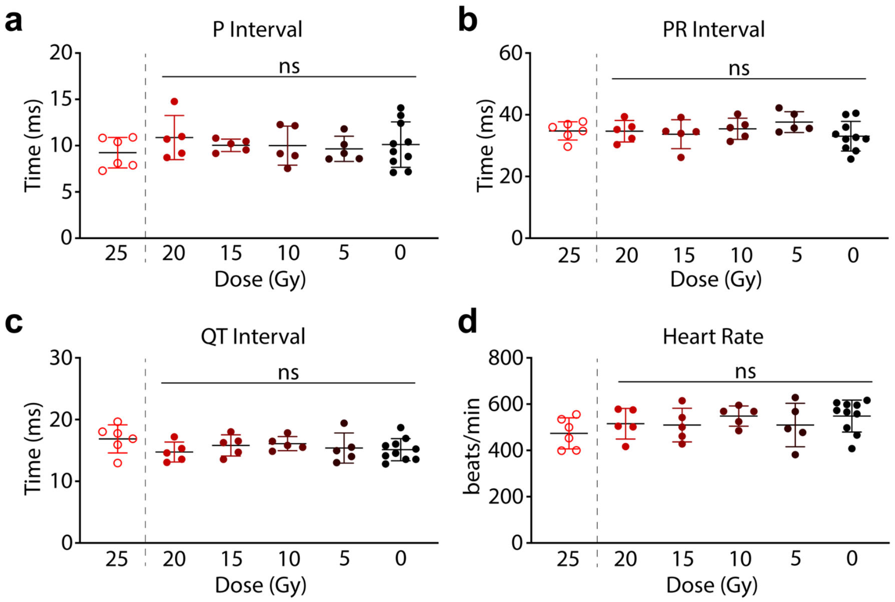

Supplementary Fig. 7. Dose-dependent effects of radiation on murine ECG intervals. **(a-d)** Dose-dependent effects of radiation on **(a)** P interval (P = 0.91), **(b)** PR interval (P = 0.38), **(c)** QT interval (P = 0.75), and **(d)** heart rate (HR, P = 0.72) at 6 weeks post IR (n = 5 biologically independent animals per IR condition; n = 10 biologically independent 0 Gy control animals). Values from an earlier twenty-five Gy cohort are plotted for reference (left, open circles, from Supplementary Fig. 4.) and were not used in statistical comparisons. Nonsignificant statistics determined by ordinary one-way ANOVA. All bar graphs are represented as mean ± SD. Source data are provided as a Source Data file.

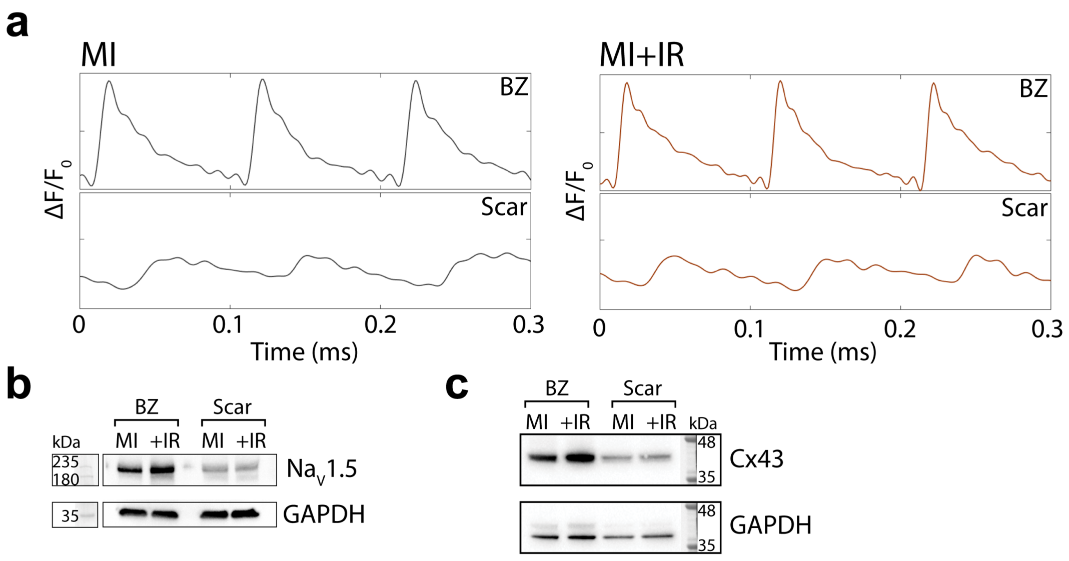

Supplementary Fig. 8. Cardiac RT in a murine model of large myocardial infarction. (**a**) Representative OAPs from MI (left) and MI+IR (right) hearts in BZ myocardium (top) and scar regions (bottom) during S1 ventricular stimulation. Optical signal from BZ exhibit OAPs with large-magnitude, fast depolarization and slow repolarization, while scar regions exhibit small electrostatic deflections, and low peak optical voltage (<50% BZ peak optical voltage), due to isodepolarization of nearby BZ myocardium. (**b** and **c)** Representative Western blots comparing $Na_V1.5$ and Cx43 protein in border zone versus scar zone myocardium in MI and MI+IR murine hearts demonstrating less conduction proteins in scar compared to BZ. Western blot experiments were replicated three times in biologically independent samples and produced similar results.

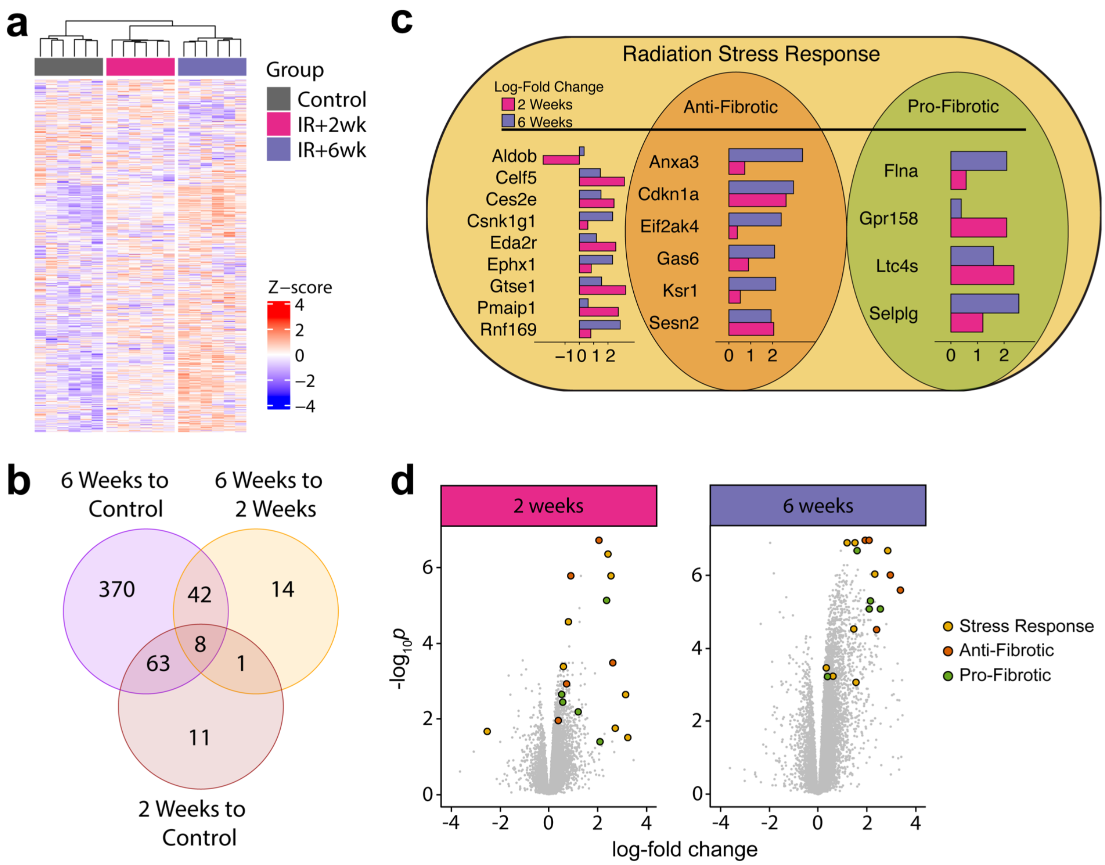

Supplementary Fig. 9. Post-IR states are differentiated by gene expression changes. (a) Heat map of all 509 differentially expressed genes at 2 weeks and 6 weeks post-IR. Samples are grouped by hierarchical clustering of gene states. (**b)** Venn-diagram of differentially-expressed genes at 2 weeks and 6 weeks post-IR across all comparisons. Gene expression changes present at 2 weeks tended to remain present at 6 weeks, and greatest number of expression changes were observed comparing control and 6-week time points. **(c)** Log-fold expression changes in radiation stress response genes. Genes are grouped by their potential pro- and anti-fibrotic activity based on prior publications (Supplementary Table 2). **(d)** Volcano plots showing fold-change and significance for all genes. Stress response genes from panel (c) are highlighted.

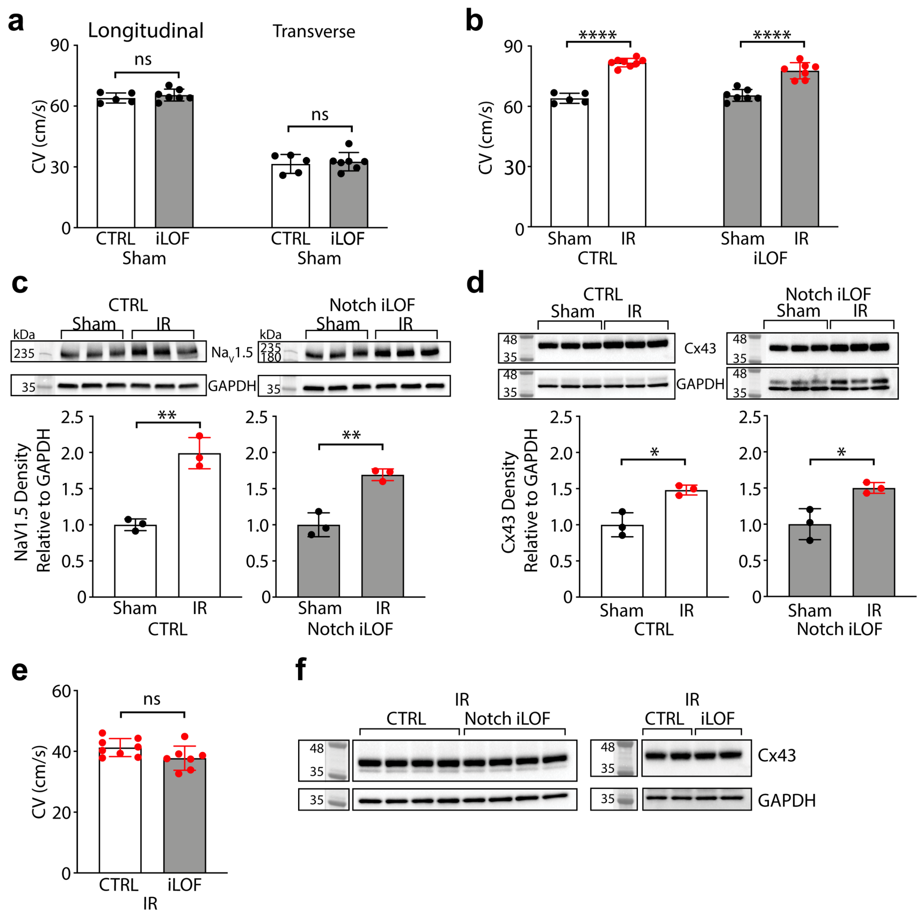

Supplementary Fig. 10 Loss of cardiomyocyte Notch signaling partially rescues radiation-induced electrical reprogramming. (a) Conduction velocities of nonirradiated (sham) littermate control (CTRL, white bar, $n = 5$ biologically independent animals) and Notch iLOF (grey bar, $n = 7$ biologically independent animals) hearts reveal no baseline difference in conduction (Longitudinal $P = 0.38$; Transverse $P = 0.69$). **(b)** Longitudinal conduction velocities in sham (black points) versus IR (red dots) CTRL (sham, $n = 5$ biologically independent animals; IR, $n = 8$ biologically independent animals; $^{****}P < 0.0001$) and Notch iLOF (sham, $n = 7$ biologically independent mice, versus IR, $n = 7$ biologically independent mice, $^{****}P < 0.0001$) mice. Values from Figure 7b and Supplementary Figure 10a have been replotted for sham versus IR comparisons. **(c)** $Na_V1.5$ in CTRL and Notch iLOF mice in the presence and

absence of radiation (*n* = 3 biologically independent samples per condition; CTRL $^{**}P = 0.0017$; iLOF $^{**}P = 0.0028$). **(d)** Cx43 in CTRL and Notch iLOF mice in the presence and absence of radiation (*n* = 3 biologically independent samples per condition; CTRL, $^{*}P = 0.010$; iLOF $^{*}P = 0.019$). **(e)** Transverse conduction velocity measurements of CTRL (*n* = 8 biologically independent animals) and Notch iLOF (n = 7 biologically independent animals) hearts at 6 weeks post-IR (*P* = 0.072). **(f)** Full Western blots of *n* = 6 biologically independent samples for Cx43 values plotted in Figure 7d. All *P* values determined by two-way unpaired *t* test. All bar graphs are represented as mean ± SD. Source data are provided as a Source Data file.

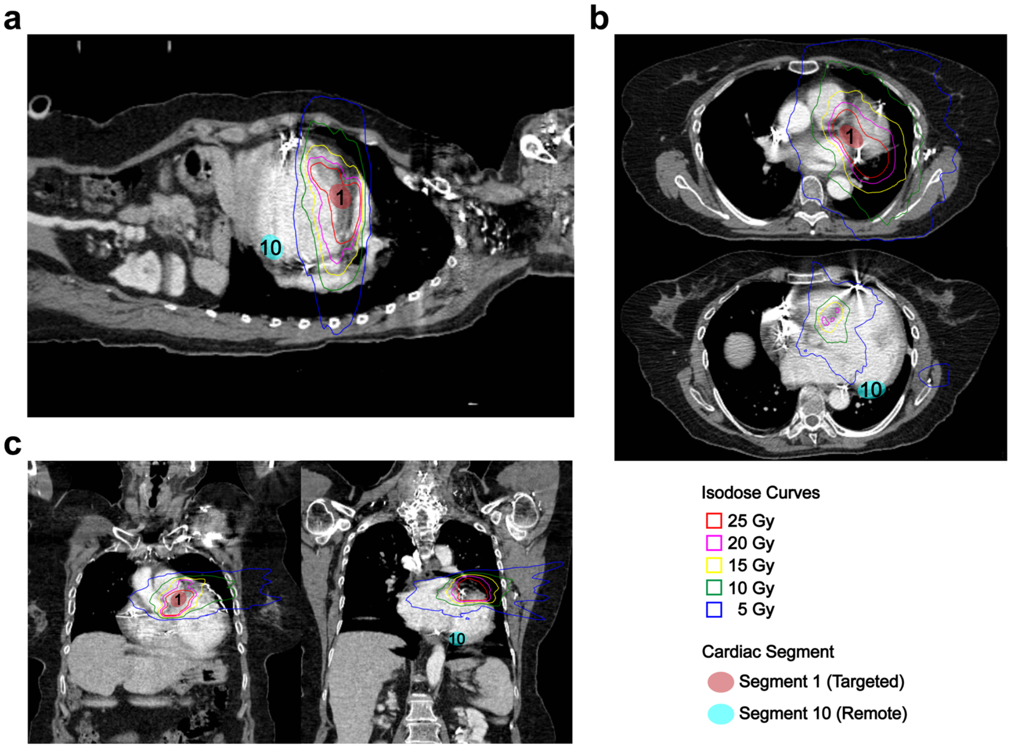

Supplementary Fig. 11. Remote, nontargeted heart specimens collected from cardiac RT patients received minimal radiation exposure. (a-c) Orthogonal views of Patient F's radiation treatment plan overlayed with isodose curves. The anterior basal LV, corresponding to Segment 1, was previously treated with 25 Gy and collected at time of explant; the posterior mid-LV, corresponding to Segment 10, was collected as a remote, nontargeted control. Segment 1 was within the 25 Gy isodose line (red), while Segment 10 was outside of the 5 Gy isodose line (blue).

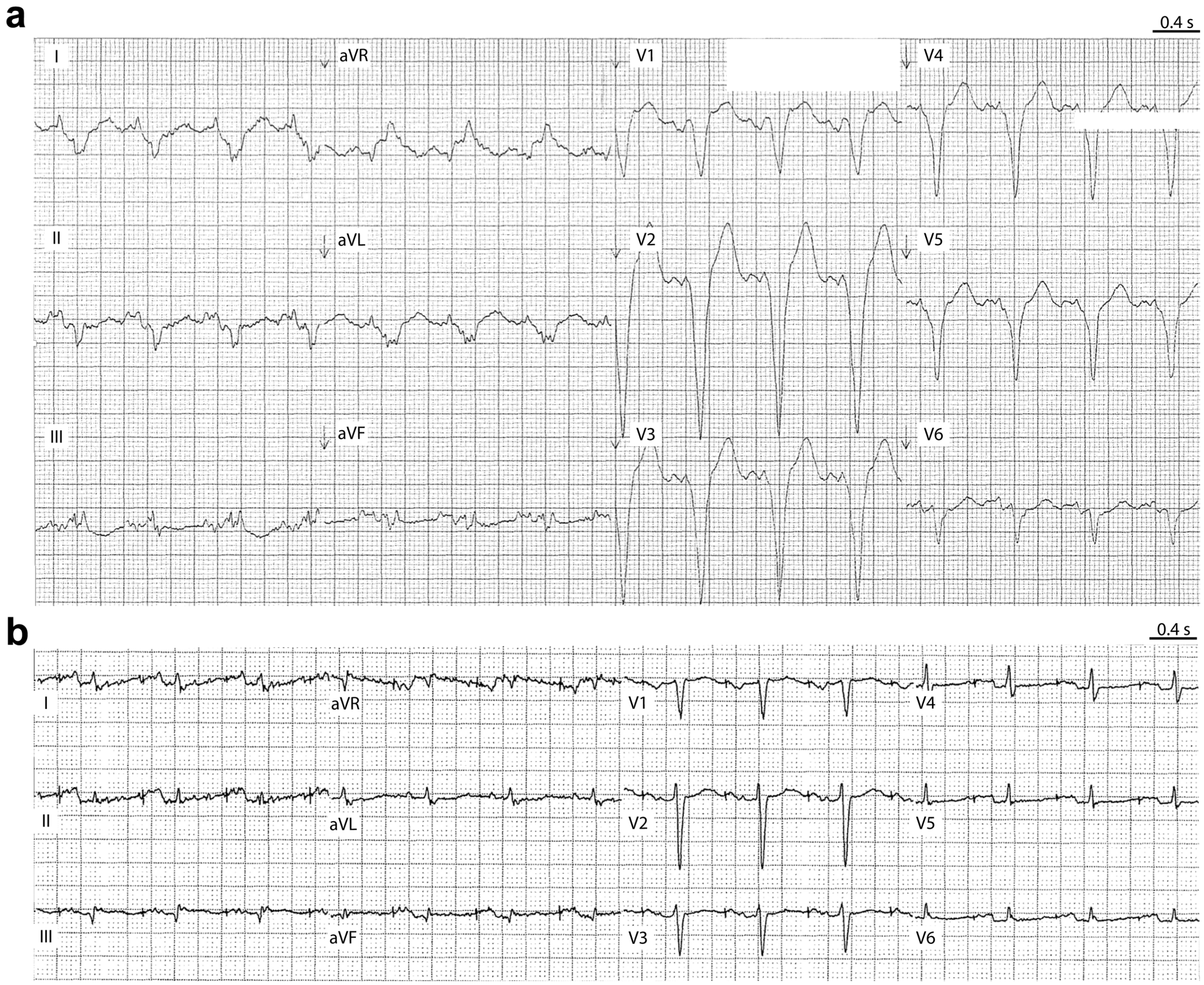

Supplementary Fig. 12. QRS shortening in Patient H after cardiac RT. (a) Twelve-lead surface ECG recorded from a Patient H at the time of RT treatment, showing a widened QRS (182 ms). (**b**) Twelve-lead surface ECG recorded from Patient H after 6 weeks post-RT, showing a shortened QRS (106 ms).

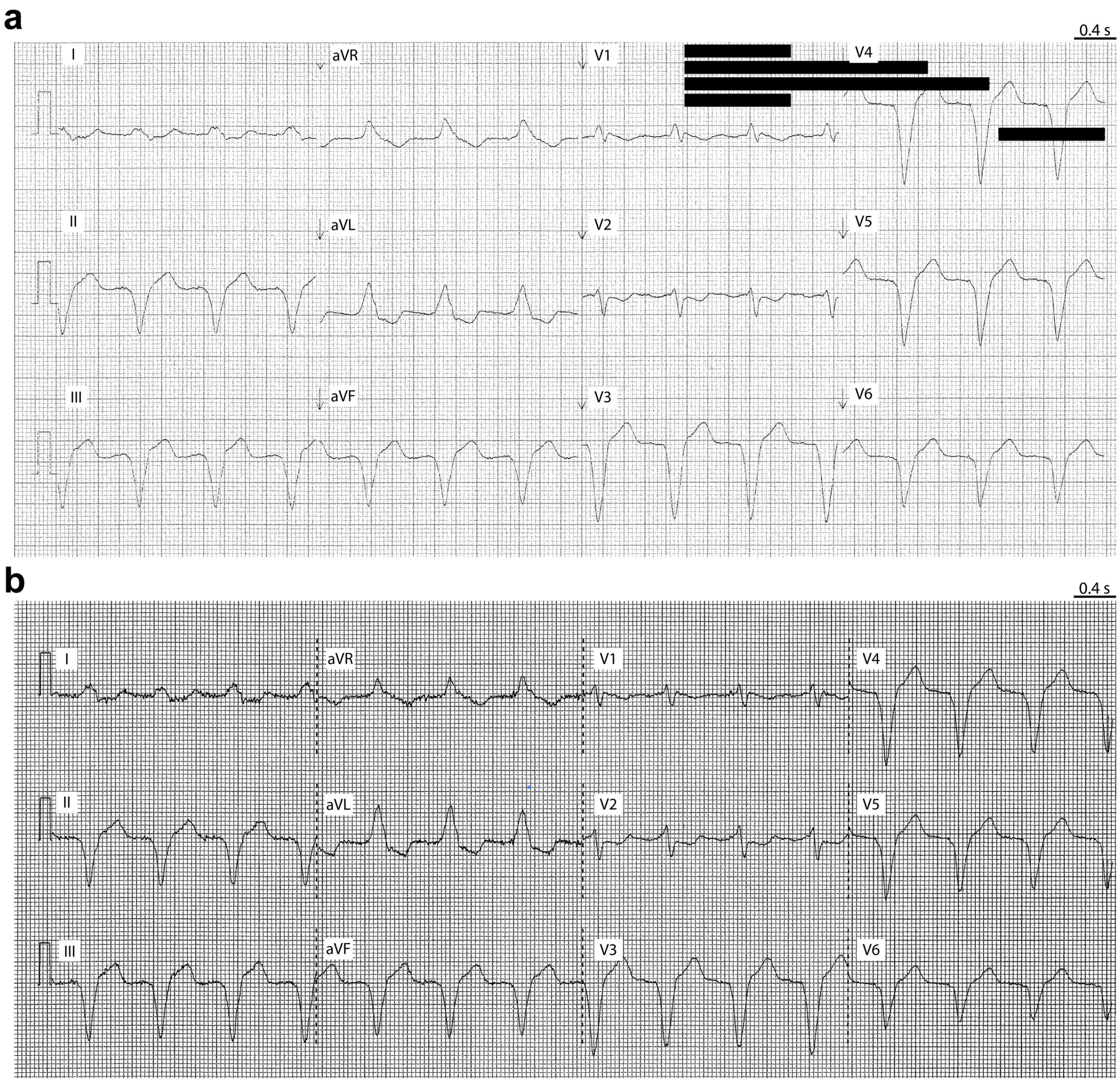

Supplementary Fig. 13. QRS shortening in Patient I after cardiac RT. (a) Twelve-lead surface ECG recorded from Patient I at the time of treatment, showing a QRS of 210 ms. (**b**) Twelve-lead surface ECG recorded from Patient I 6 weeks post-RT, showing a QRS of 185 ms.

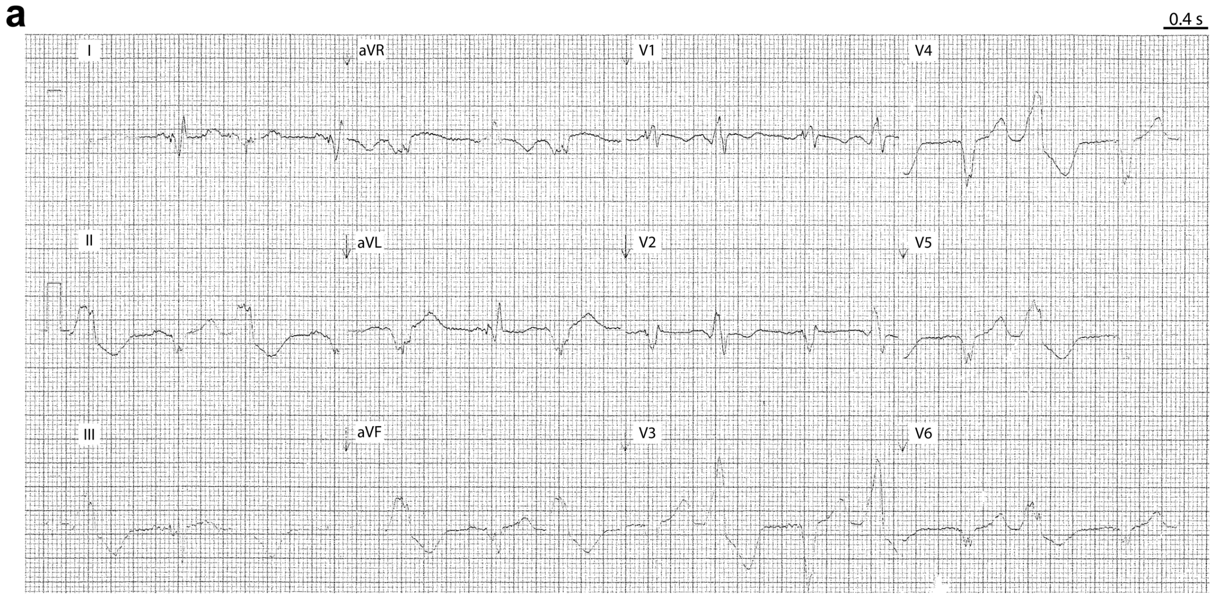

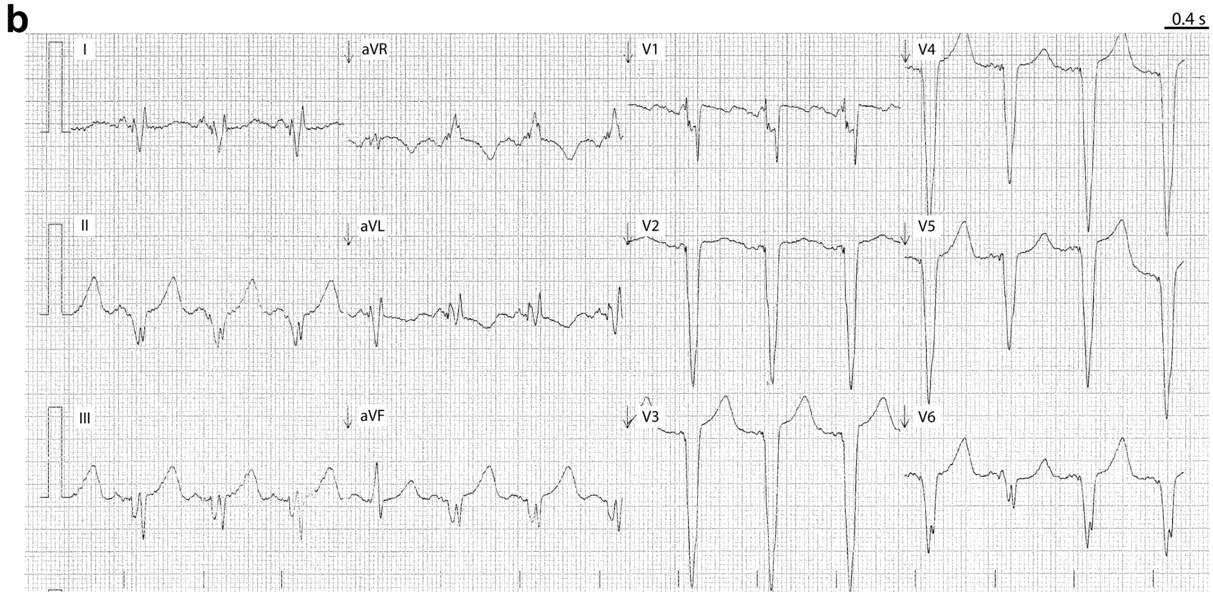

Supplementary Fig. 14. QRS shortening in Patient J after cardiac RT. (a) Twelve-lead surface ECG recorded from RT Patient J at the time of treatment, showing ventricular bigeminy and a QRS of 170 ms. (**b**) Twelve-lead surface ECG recorded from Patient J after 3 months post-RT, showing resolution of bigeminy and a shortened QRS of 145 ms.

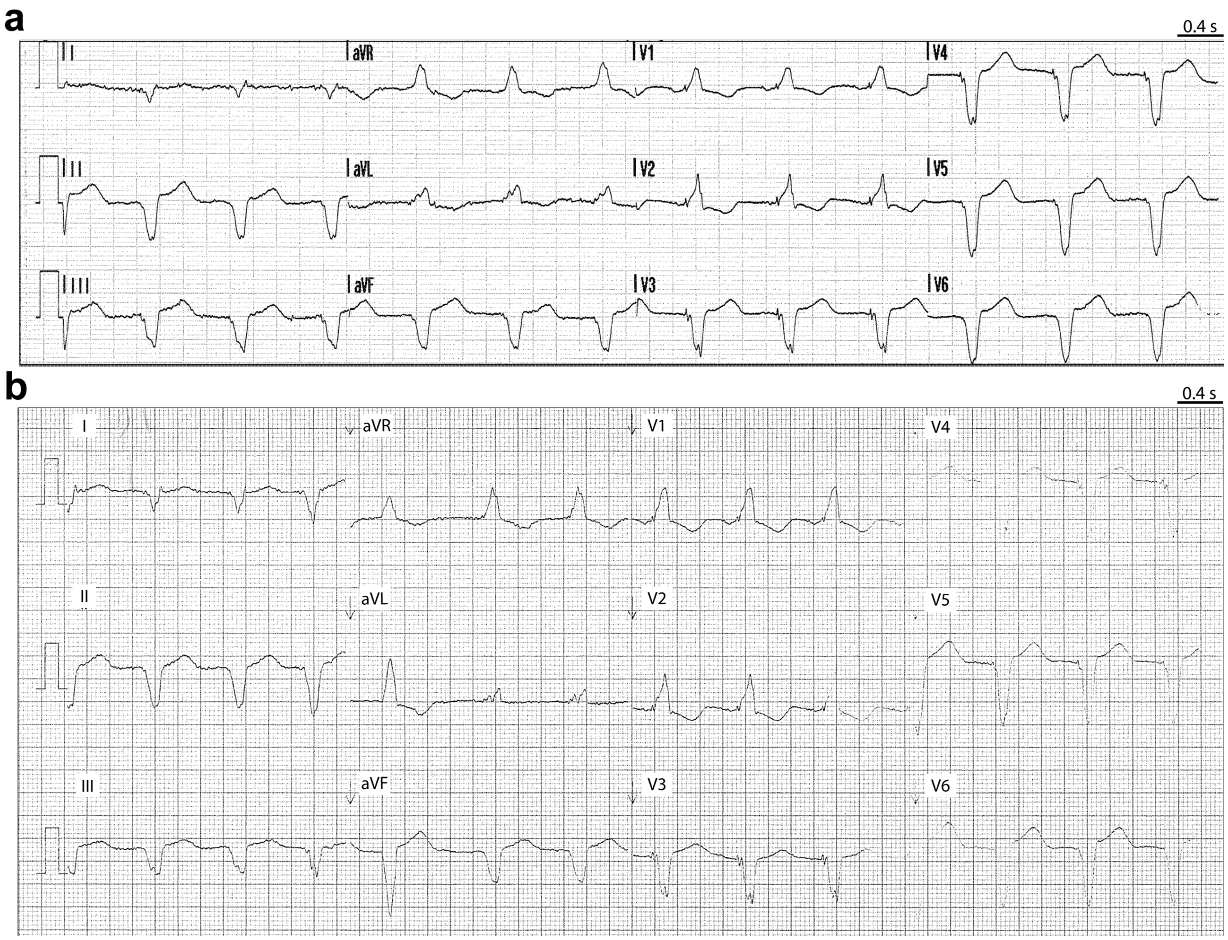

Supplementary Fig. 15. QRS intervals in Patient B after cardiac RT. (a) Twelve-lead surface ECG recorded from RT Patient B at the time of treatment, showing a baseline QRS of 180 ms. (**b**) Twelve-lead surface ECG recorded from Patient B at 6 weeks post-RT, showing a similar QRS of 175 ms.

Variable	Patients A-J
Median Age (year) [Range]	66 [49-84]
Sex (n)	
Female	2
Male	8
NYHA Class (n)	
I (1)	1
III (7)	7
IV (2)	2
Cardiomyopathy (n)	
ICM	4
NICM*	8
Median BMI [Range]	31.7 [23.8-48.6]
Comorbidities (n)	
CHF	9
COPD	1
CKD	2
CVA	2
DM	4
HD	1
MI	4
Median AADs [Range]	2 [1-2]
Median Prior CAs [Range]	2 [0-3]

Supplementary Table 1. Summary of the clinical information of cardiac RT Patients A through J included in the present study. NYHA = New York Heart Association heart failure classification; ICM = ischemic cardiomyopathy; NICM = nonischemic cardiomyopathy; BMI = body mass index; CHF = congestive heart failure; COPD = chronic obstructive pulmonary disease; CKD = chronic kidney disease; CVA = cerebrovascular accident; HD = hepatic disease; DM = diabetes mellitus; MI = myocardial infarction; AAD = anti-arrhythmic drug; CA = catheter ablation

*Includes 2 patients with NICM prior to MI

Gene	Role in Radiation Stress	Citation DOI	Role in Fibrosis	Fibrosis Citation DOI
Aldob	Suppresses G6PD activity in complex with p53	10.1038/s43018-020-0086-7	NA	NA
Anxa3	Activates JNK	10.1016/j.stemcr.2015.05.013	Inhibits PI3K/Akt	10.1002/jcp.27717
Ccl8	NA	NA	NA	NA
Cdkn1a	Anti-proliferative p53 target	10.1158/0008-5472.CAN-04-3995	Blocks angiotensin II	10.1016/j.peptides.2016.07.003
Celf5	p53 target		NA	NA
Ces2e	p53 target	10.1016/j.ebiom.2017.05.017	NA	NA
Csnk1g1	Inhibits p53 phosphorylation	10.1186/1476-4598-13-231	NA	NA
Eda2r	p53 target, binds extodysplasin EDA-A2	10.1126/science.290.5491.523	NA	NA
Eif2ak4	Induces p21 expression	10.1371/journal.pgen.1005212	Reduces collagen deposition	10.1161/JAHA.116.004453
Ephx1	NA	NA	NA	NA
Flna	BRCA scaffold	10.1530/ERC-13-0364 jk	ECM production	10.1536/ihj.17-446
Gas6	AXL ligand	doi:10.1172/JCI85610	Anti-apoptotic	10.1016/j.exger.2019.01.029
Gpr158	PI3K-AKT activation after DNA damage	10.1038/nrd3320	Increases collagen deposition	10.1161/CIRCGENETICS.114.000537

Supplementary Table 2. Top genes by adjusted P-value (limited to log-fold change >2) for comparisons of 2 weeks post-IR versus control and 6 weeks post-IR versus control and their roles in radiation stress and fibrosis.
NA = not available; DOI = digital object identifier

Target	Forward Primer	Reverse Primer	Length (bp)
rtTA	GGACGAGCTCCACTTAGACGGCGAGGACGT	TCTGCTCAAACTCGAAGTCGGCCATATCC	170
tetO-NICD	TTTTGACCTCCATAGAAGACACCGGGACCG	CTGACACTTTGAAACCCTCAGGGAACCAG	210
Cre	GCATTACCGGTCGATGCAACGAGTGATGAG	GAGTGAACGAACCTGGTCGAAATCAGTGCG	408
R26r-wt	CAAAGTCGCTCTGAGTTGTTATCAGTAAGG	GGAGCGGGAGAAATGGATATGAAGTACTGG	486
R26r-mut	CAAAGTCGCTCTGAGTTGTTATCAGTAAGG	TCCAAGAGTACTGGAAAGACCGCGAAGAGT	332

Supplementary Table 3. Primers and predicted product lengths of targets used for detection and genotyping of transgenic mice. rtTA = reverse tetracycline-controlled transactivator; tetO-NICD = tetracycline-On-Notch IntraCellular Domain; R26r-wt = wild-type ROSA26; R26r-mut = mutant ROSA26 expressing dnMAML.
