# Supplementary material for: Cardiac radiotherapy induces electrical conduction reprogramming in the absence of transmural fibrosis
Source: Nat Commun. 2021 Sep 24;12:5558. doi: 10.1038/s41467-021-25730-0 (PMC8463558; doi:10.1038/s41467-021-25730-0)
Supplement: Supplementary file 3 — Reporting Summary [file 41467_2021_25730_MOESM3_ESM.pdf]

nature research

Corresponding author(s): Stacey L. Rentschler, M.D., Ph.D.

Last updated by author(s): 8/17/2021

Reporting Summary

Nature Research wishes to improve the reproducibility of the work that we publish. This form provides structure for consistency and transparency in reporting. For further information on Nature Research policies, see our Editorial Policies and the Editorial Policy Checklist.

Statistics

For all statistical analyses, confirm that the following items are present in the figure legend, table legend, main text, or Methods section.

n/a	Confirmed	
☐	☒	The exact sample size (*n*) for each experimental group/condition, given as a discrete number and unit of measurement
☐	☒	A statement on whether measurements were taken from distinct samples or whether the same sample was measured repeatedly
☐	☒	The statistical test(s) used AND whether they are one- or two-sided<br>*Only common tests should be described solely by name; describe more complex techniques in the Methods section.*
☒	☐	A description of all covariates tested
☐	☒	A description of any assumptions or corrections, such as tests of normality and adjustment for multiple comparisons
☐	☒	A full description of the statistical parameters including central tendency (e.g. means) or other basic estimates (e.g. regression coefficient) AND variation (e.g. standard deviation) or associated estimates of uncertainty (e.g. confidence intervals)
☐	☒	For null hypothesis testing, the test statistic (e.g. *F*, *t*, *r*) with confidence intervals, effect sizes, degrees of freedom and *P* value noted<br>*Give P values as exact values whenever suitable.*
☒	☐	For Bayesian analysis, information on the choice of priors and Markov chain Monte Carlo settings
☒	☐	For hierarchical and complex designs, identification of the appropriate level for tests and full reporting of outcomes
☒	☐	Estimates of effect sizes (e.g. Cohen's *d*, Pearson's *r*), indicating how they were calculated

Our web collection on statistics for biologists contains articles on many of the points above.

Software and code

Policy information about availability of computer code

Data collection	Libraries were sequenced with the Illumina NovaSeq S4. Murine ECGs were recorded using the commercially-available ECG software in LabChart (v.8.0, ADInstruments). Optical mapping data was collected through through the MiCAM Ultimate software (v.2011.11, BrainVision LLC). All Western blot image collection was performed using Image Lab (v.6.0, Bio-Rad). Colorimetric data collection was performed using Gen5 software (v.2.0.4, Agilent BioTek).
Data analysis	Prism (v.8.02, GraphPad Software Inc.) was used to perform statistical analyses and plot data. Power calculations were performed using G*power (v.3.1.9).<br>RNA sequencing reads were aligned to the Mus musculus GRCm38.76 genome using STAR (v. 2.7.3a.), and alignment quality metrics were obtained from RNA-SeQC (v. 2.3.5). Transcript abundance was quantified in Salmon (v. 1.0), and downstream analysis utilizing previously-published algorithms (detailed in Methods) was performed in R (v. 3.6.3).Transcript abundance was summarized to gene counts using tximport (v. 3.10). Gene counts were scaled to library size using trimmed mean of M-value (TMM) normalization in EdgeR (v. 3.28). Weighted likelihoods were calculated and differential expression analysis performed in limma (v. 3.42). Pathway analysis and gene set enrichment analysis were performed in GSEA (v. 4.1.0).<br>Fibrosis quantification was performed in MATLAB (2017b, MathWorks) using previously-published methods (see Methods). ECG interval analysis was performed using the commercially-available ECG software in LabChart (v.8.0, ADInstruments). Optical mapping analysis was performed on MATLAB using open source Rhythm software (Rhythm2014b, http://efimovlab.org/research/resources/rhythm). All Western blot densitometry was performed using Image Lab (v.6.0, Bio-Rad). Colorimetric analysis was performed using Gen5 software (v.2.0.4 Agilent BioTek) and Excel (v.16.52, Microsoft). All murine immunohistochemistry quantification was performed using ImageJ (v.2.0.0, NIH).

For manuscripts utilizing custom algorithms or software that are central to the research but not yet described in published literature, software must be made available to editors and reviewers. We strongly encourage code deposition in a community repository (e.g. GitHub). See the Nature Research guidelines for submitting code & software for further information.

Data

Policy information about availability of data

All manuscripts must include a data availability statement. This statement should provide the following information, where applicable:

- Accession codes, unique identifiers, or web links for publicly available datasets
- A list of figures that have associated raw data
- A description of any restrictions on data availability

All the data supporting the findings from this study are available within the manuscript and its supplementary information. Transcriptomic data that support the findings of this study have been deposited with GEO under accession number GSE153981[https://www.ncbi.nlm.nih.gov/geo/query/acc.cgi?acc=GSE153981]. Annotated gene sets were obtained from the Molecular Signatures Database (MSigDB) [https://www.gsea-msigdb.org/gsea/msigdb/genesets.jsp?collection=H]. Source data are provided with this paper.

Field-specific reporting

Please select the one below that is the best fit for your research. If you are not sure, read the appropriate sections before making your selection.

☒ Life sciences ☐ Behavioural & social sciences ☐ Ecological, evolutionary & environmental sciences

For a reference copy of the document with all sections, see nature.com/documents/nr-reporting-summary-flat.pdf

Life sciences study design

All studies must disclose on these points even when the disclosure is negative.

Sample size	Sample size for each murine experimental cohort was based on a t-test to test for differences in conduction velocity or effective refractory period. A study with an effect size of 0.25 and a power of 80% required a minimum of n = 6 independent biological replicates per group, to test this difference at 5% significance using a two-tailed test. Both male and female littermates were used for all experiments. For experiments involving aged or surgery mice we overpowered these cohorts in anticipation of procedure-related death.<br>For Western blot on samples obtained from wild-type irradiated and iNICD mice, a study with an effect size of 0.80 and power of 80% required a minimum of n = 3 independent biological replicates per group to test this difference at 5% significance using a two-tailed test. In anticipation of a smaller effect size for partial rescue experiments in Notch iLOF experiments, a study was designed instead to test for an effect size of 0.25 and power of 80%, and these required a minimum of n = 6 independent biological replicates per group to test for differences at 5% significance using a two-tailed test. This sample size was subsequently used to power a test for differences in Cx43 expression in iNICD mice after a Referee commented that effect sizes appeared lower for Cx43 compared to Nav1.5.<br>For RNA sequencing, an n = 6 biologically independent samples per treatment condition was chosen to detect meaningful transcriptional changes in heart tissue based on previous publications.<br><br>In patients, sample size was determined by ENCORE-VT (see below), a single-arm Phase I/II clinical trial, in which 19 patients were enrolled. This sample size was initially chosen to demonstrate acute safety and preliminary efficacy of cardiac radiation therapy for refractory VT treatment as a Phase I/II trial. All patient specimens and samples were included, when available, due to scarcity of this resource.
Data exclusions	No data was excluded in this study.
Replication	For immunohistochemistry and micrographs shown, experiments were repeated a minimum of 3 times using biologically independent specimens (in murine studies) or 2 times in technically independent replicates on the same patient specimens (in human studies). All attempts at replication produced similar results.<br>Immunoblotting experiments were repeated to a minimum of 3 technical triplicates to replicate findings and all attempts at replication produced similar results.<br>Electrophysiologic experiments on irradiated healthy and infarcted wild-type mice at 6 weeks post-irradiation were each performed twice independently using biologically independent cohorts. All attempts at replication produced similar results. Long-term experiments (wild type 42-week and Notch iNICD/iLOF transgenic 12-52-week) were each performed once due to time.
Randomization	For all murine experiments, male and female mice were randomized to treatment/non-treatment groups prior to experimental treatment.<br>In patients, data was collected from a study designed as a single-arm Phase I/II clinical trial (ENCORE-VT, see below) to evaluate safety and preliminarily assess efficacy of radiation therapy for VT management; thus, all enrolled patients received treatment. Although single-arm studies are inherently limited by the lack of controls and randomization, patient-level data were restricted to paired, binary intra-patient comparisons (non-targeted versus targeted and/or pre-treatment versus post-treatment).
Blinding	All investigators were blinded to sample treatment allocation during sample collection, data collection and analysis.

Reporting for specific materials, systems and methods

We require information from authors about some types of materials, experimental systems and methods used in many studies. Here, indicate whether each material, system or method listed is relevant to your study. If you are not sure if a list item applies to your research, read the appropriate section before selecting a response.

Materials & experimental systems

n/a	Involved in the study	
☐	☒	Antibodies
☒	☐	Eukaryotic cell lines
☒	☐	Palaeontology and archaeology
☐	☒	Animals and other organisms
☐	☒	Human research participants
☐	☒	Clinical data
☒	☐	Dual use research of concern

Methods

n/a	Involved in the study	
☒	☐	ChIP-seq
☒	☐	Flow cytometry
☒	☐	MRI-based neuroimaging

Antibodies

Antibodies used

All primary antibodies used: Anti-Phospho-Histone H2AX (R&D Systems AF2288, lot KNH1218111); Anti-Nav1.5 (Alomone Labs, 493-511, lot ASC005AN3002 and ASC005AN3702); Anti-Connexin43 (Invitrogen, 71-0700, lot PI209083); Anti-N-cadherin (Invitrogen, 33-3900, lot UC284646).); Anti-GAPDH (Cell Signaling Technology 14C10, lot 14).

Secondary antibodies used: Alexa 568 (Abcam ab175471, lot 1494753); Alexa 488 (Abcam ab150077, lot 3244688) and Alexa 488 (Abcam ab150121, lot 3201847), Anti-Rabbit HRP (Abcam ab6721, lot GR3321356).

Validation

Anti-Phospho-Histone H2AX (R&D Systems AF2288) validation was stated on the manufacturer's website and confirmed for IHC in Mus musculus as described previously [Jangi et al., SMN deficiency in severe models of spinal muscular atrophy causes widespread intron retention and DNA damage. PNAS, 2017;114(12):E2347-E2356] [Wang et al., BRCA1 intronic Alu elements drive gene rearrangements and PARP inhibitor resistance. Nature Communications, 2019;10:5661].
Anti-Nav1.5 (Alomone Labs, 493-511) validation was stated on the manufacturer's website and confirmed for WB and IHC in Homo sapiens and Mus musculus as described previously [Salvarani et al., The K219T-Lamin mutation induces conduction defects through epigenetic inhibition of SCN5A in human cardiac laminopathy. Nature Communications, 2019;10:2267] [de la Roche et al., Comparing human iPSC-cardiomyocytes versus HEK293T cells unveils disease-causing effects of Brugada mutation A735V of Na V 1.5 sodium channels. Scientific Reports, 2019;9(1):11173] [Li et al. Differential Wnt-mediated programming and arrhythmogenesis in right versus left ventricles. JMCC, 2018;132;92-107].
Anti-Connexin43 (Invitrogen, 71-0700) validation was stated on the manufacturer's website and confirmed for WB and IHC in Homo sapiens and Mus musculus as described previously [Roberts, Murphy, et al., Ankyrin-B dysfunction predisposes to arrhythmogenic cardiomyopathy and is amenable to therapy. Journal of Clinical Investigation, 2019;129(8):3171-3184] [Li et al. Differential Wnt-mediated programming and arrhythmogenesis in right versus left ventricles. JMCC, 2018;132;92-107] [Nguyen, et al. Engineering prokaryotic channels for control of mammalian tissue excitability. Nature Communications, 2016;7:13132.]
Anti-N-cadherin (Invitrogen, 33-3900) validation was stated on the manufacturer's website and confirmed for IHC in Homo sapiens and Mus musculus as described previously Roberts, Murphy, et al., Ankyrin-B dysfunction predisposes to arrhythmogenic cardiomyopathy and is amenable to therapy. Journal of Clinical Investigation, 2019;129(8):3171-3184] [Li et al. Differential Wnt-mediated programming and arrhythmogenesis in right versus left ventricles. JMCC, 2018;132;92-107].
Anti-GAPDH (Cell Signaling Technology 14C10) validation was stated on the manufacturer's website and confirmed for WB in Homo sapiens and Mus musculus as described previously [Li et al. Differential Wnt-mediated programming and arrhythmogenesis in right versus left ventricles. JMCC, 2018;132;92-107] [Dan et al., DNA damage invokes mitophagy through a pathway involving Spata18. Nucleic Acids Research, 2020;48(12)6611-6623.]

Animals and other organisms

Policy information about studies involving animals; ARRIVE guidelines recommended for reporting animal research

Laboratory animals

All mice were housed in 12-hour light/dark cycles, at ambient temperatures of 20-22 degrees Celsius, at a humidity range between 40-60%, and with access to food and water ad libitum, in accordance with animal study guidelines at Washington University.

Outbred adult male and female littermate CD-1 mice were purchased (Charles River Laboratories, Wilmington MA). iNICD (MHCrtTA and tetO_NICD) and Notch LOF (Myh6-MerCreMer and R26R-DNMAML/+) mice have been described previously and were maintained on a mixed genetic background. In all experiments,both male and female mice aged 8 weeks or greater were used, and age- and gender-matched littermate control animals were used for comparison.

Wild animals

This study did not involve wild animals.

Field-collected samples

This study did not involve field-collected samples.

Ethics oversight

Animal protocols were approved by the Animal Studies Committee at Washington University School of Medicine.

Note that full information on the approval of the study protocol must also be provided in the manuscript.

Human research participants

Policy information about studies involving human research participants

Population characteristics

Patients with refractory ventricular tachycardia (VT) (i.e. failed at least one catheter ablation or was contraindicated to

	catheter ablation and failed/became intolerant to at least one anti-arrhythmic medication) were included into the ENCORE-VT study (NCT02919618). Any patient that met inclusion/exclusion criteria for the study and agreed to consent received Stereotactic Body Radiotherapy (SBRT) to the heart regardless of sex/gender, race, and ethnicity. However, sex/gender, race, and ethnicity were documented in the EDC. Because VT can occur in all sexes/genders, races, and ethnic groups, all patients regardless of sex/gender, race, or ethnic group were eligible for inclusion in this study.<br><br>All patients signed an IRB approved Informed Consent Form prior to SBRT and collection of specimens. A total of 21 participants signed the consent form. Two of these participants screen failed prior to SBRT and the remaining 19 participants were treated. Of these 19 participants, 17 were male and 2 were female. Of these 19 participants, 17 were white, 1 was African American, and 1 was Asian. None were Hispanic/Latino.
Recruitment	Subjects were recruited through the cardiology clinic and hospital. The cardiology PI would identify a potentially eligible patient during either a clinic visit or hospital stay. He would then discuss with the radiation oncology PI who would also meet with the patient to discuss the study and determine eligibility. If the patient was deemed eligible by both physicians, the patient would then be enrolled by one of the physicians or a research coordinator.<br><br>Because inclusion criteria included refractory VT (i.e. failed at least one catheter ablation, was contraindicated to catheter ablation and failed/became intolerant to at least one anti-arrhythmic medication, and had greater than or equal to 3 VT episodes over 6 months), there likely exists a narrow patient selection bias that prohibits generalization of results to a larger population. Additionally, survival bias limits complete understanding of treatment benefits and risks. This study is inherently limited by its single-arm design, low number of patients recruited, and recruitment and treatment conducted at a single academic center. Treatment currently remains investigational.
Ethics oversight	Washington University Institutional Review Board

Note that full information on the approval of the study protocol must also be provided in the manuscript.

Clinical data

Policy information about clinical studies

All manuscripts should comply with the ICMJEguidelines for publication of clinical research and a completedCONSORT checklist must be included with all submissions.

Clinical trial registration	The study reported in this manuscript reports clinical data collected from the following clinical trial: NCT02919618
Study protocol	The protocol for the Phase I/II Study of EP-guided Noninvasive Cardiac Radioablation for Treatment of Ventricular Tachycardia (ENCORE-VT) study can be accessed on clinicaltrials.gov.
Data collection	Patients were recruited at Washington University between July 2016 and December 2017. Data on enrolled participants was collected from July 2016 to present, as we are still collecting data as part of the extended follow up period of the study. This extended follow up period should end in approximately January 2023.<br><br>Data was collected from both the participants' medical records, as well as documented on paper source documents. This data was then entered into and stored in REDCap on Washington University's servers. Use of REDCap at Washington University is supported by Clinical and Translational Science Award (CTSA) Grant [UL1 TR000448] and Siteman Comprehensive Cancer Center and NCI Cancer Center Support Grant P30 CA091842.
Outcomes	Original Study Objectives:<br>Primary Objectives<br>1. Phase I - Demonstrate acute (≤ 90 days) safety of noninvasive stereotactic cardiac ablation radiotherapy (ENCORE). The primary safety endpoint is defined by a ≤ 20% rate of serious adverse events (SAEs) using CTCAE v4.0 criteria that are possibly/probably/definitely related to study treatment, based on previously published data for expected invasive catheter-based VT-ablation procedures.<br>2. Phase II - Demonstrate preliminary efficacy of ENCORE. The primary efficacy endpoint is defined by the number of subjects with a reduction in ICD therapies (ATP and ICD shocks) comparing the period six months before ENCORE treatment to the six months after ENCORE treatment as adjudicated by continuous ICD monitoring. There will be a six-week "blanking period" after therapy to allow for ablation effect. For patients with PVC-induced cardiomyopathy, the primary efficacy will be any reduction in PVC burden based on ambulatory heart monitors.<br><br>Secondary Objectives<br>1. Determine six-month and twelve-month survival (overall mortality endpoint) after treatment with ENCORE.<br>2. Determine late toxicity endpoint (>90 days to 12 months), as tracked prospectively after treatment using CTCAE v4.0 criteria.<br>3. Determine patient-reported health related quality of life endpoint (HRQOL) as measured by changes between pre-treatment and 6-week, 6-month, and 12-month post treatment scores on the standardized SF-36 questionnaire.<br>4. Evaluate stricter efficacy endpoint of ENCORE treatment, as defined by number of patients who have had 50% reduction in any VT therapies (ATP or ICD shocks) after ENCORE treatment (6 months before vs. 6 months after treatment, with a 6 week blanking period immediately after treatment). For patients with PVC-induced cardiomyopathy, the stricter efficacy will be >50% reduction in PVC burden based on ambulatory heart monitors.<br>5. Evaluate strictest efficacy endpoint of ENCORE treatment, as defined by number of patients who have had 95% reduction in any VT (ATP or ICD shocks) after ENCORE treatment (6 months before vs. 6 months after treatment, with a 6 week blanking period immediately after treatment). For patients with PVC-induced cardiomyopathy, the strictest efficacy will be abolition of PVC burden (<1%) based on ambulatory heart monitors.<br>6. Evaluate the most clinically useful efficacy endpoint of ENCORE treatment, namely, number of patients with reduction specifically in ICD shocks (6 months before vs. 6 months after treatment, with a 6 week blanking period immediately after treatment). For

patients with PVC-induced cardiomyopathy, the most clinically useful efficacy will be improvement in cardiac function in the setting of any improvement in PVC burden.
7. Evaluate longer-term durability endpoint of ENCORE treatment, as defined by number of patients with reduction in VT therapies (ATP or ICD shock and ICD shock alone) during the early phase (treatment to 6 months, with 6 week blanking period) vs. the late phase (6 months to 1 year). For patients with PVC-induced cardiomyopathy, the longer-term durability efficacy will be persistence of any reduction in PVC burden based on ambulatory heart monitors during early phase vs. late phase.

Exploratory Objectives
1. To better understand the mechanisms and timing of radiotherapy injury, we will obtain serum blood markers of myocardial injury (troponin), endothelial injury (E-selectin), fibrosis (galectin-3) and prothrombotic markers (von Willibrand factor) at baseline, 3 days and 3 months after ENCORE treatment.
2. Evolution of electrical remodeling as obtained with noninvasive ECGI from baseline to 3 months and 12 months after ENCORE treatment
3. To better understand the effect of radiotherapy on edema, fibrosis, cardiac inflammation, cardiac metabolism and localized cardiac function, we plan to use serial cardiac imaging (DE-cMRI when possible, FDG-PET in all) at baseline, 3 days and 3 months after ENCORE treatment

Additional Analyses Outcomes:
A separate project was approved by the Washington University IRB to complete additional analyses on the original project.

These additional analyses include:
1) Descriptive analysis of baseline clinical, laboratory, imaging, and radiation plan data
2) Descriptive analysis of changes in clinical, laboratory, and imaging data
3) Association of clinical, laboratory, imaging, and radiation plan data with patient outcomes, including, but not limited to, 1) arrhythmia burden, 2) toxicity, 3) changes in laboratory values, and 4) changes in imaging features (including radiomic signatures)
4) Analysis of changes in various biomarkers including, but not limited to, cytokines and miRNAs.
